# Formation of Periodic Surface Structures by Multipulse Femtosecond Laser Processing of Au-Coated Ni in Various Fluids

**DOI:** 10.1021/acsaenm.3c00070

**Published:** 2023-04-04

**Authors:** Niusha Lasemi, Gerhard Liedl, Günther Rupprechter

**Affiliations:** † Institute of Materials Chemistry, Technische Universität Wien, 1060 Wien, Austria; ‡ Institute of Production Engineering and Photonic Technologies, Technische Universität Wien, 1060 Wien, Austria

**Keywords:** periodic surface structures, femtosecond laser processing, scanning electron microscopy, X-ray diffraction, Raman spectroscopy, contact angle

## Abstract

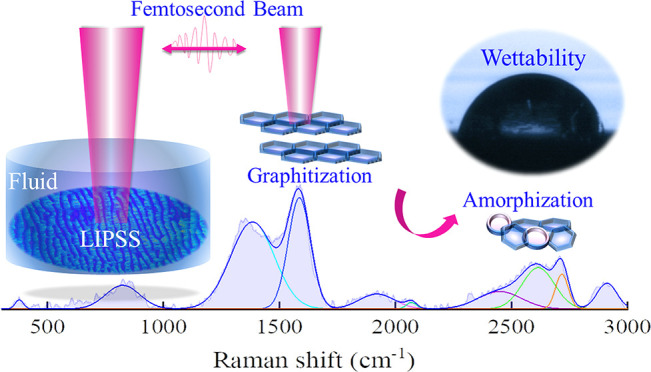

Using multipulse linearly polarized femtosecond laser processing of a Au-coated Ni surface in various liquid media created subwavelength self-organized nanoripples. The thin gold film improved the laser absorptivity, decreasing the ripple generation threshold in liquids. High spatial frequency ripples exhibited lower angular deviation than low spatial frequency ones, but in water the deviation was comparable for both types of ripples. The initiation of nanoripples may precede nanoparticle generation, which is why in hexane several cuboid Au particles were trapped between the ripples. Fast cooling processes freeze ejected molten droplets during the phase explosion and surface reorganization. Grazing incidence X-ray diffraction of samples processed in butanol showed a small shift toward smaller angles for the Ni phase, indicating a lattice expansion due to higher tensile stress. Confocal micro-Raman spectroscopy detected surface graphitization and amorphization in areas laser-treated in ethanol, butanol, and hexane, with the highest carbonization observed in butanol. Presumably, femtosecond laser-induced photolysis triggers the formation of graphite nanocrystallites, and consecutive pulses cause amorphization. Static contact angle measurements showed a general tendency toward hydrophobicity with highest contact angles for rippled areas created in butanol.

## 1 Introduction

Laser ablation can be employed for nanoparticle generation and surface structuring, both being useful in a variety of applications.^1−7^ Femtosecond laser surface engineering via generation of laser-induced periodic surface structures (LIPSS) has been frequently studied. Based on the nature of the material (target) and characteristics of the laser beam, various micro- and nanostructures can be produced which modify the mechanical, optical, and chemical properties of the material surface. Such modified surfaces can be utilized for various applications in medicine, electronics, and optics.^8−14^ Overall, two types of LIPSS exist, i.e. low spatial frequency LIPSS (LSFL) and high spatial frequency LIPSS (HSFL), that can be classified based on the ratio of LIPSS periodicity (Λ) to the laser wavelength (λ).^1^ The LSFL periodicity is close to the wavelength of the light (λ/2 ≤ Λ_LSFL_ ≤ λ), whereas the HSFL periodicity is less than half of the wavelength (Λ_HSFL_< λ/2). In special cases, so-called ultrahigh spatial frequency LIPSS (UHSFL) can be formed with a periodicity below 100 nm.^15−17^

**Figure 1:**
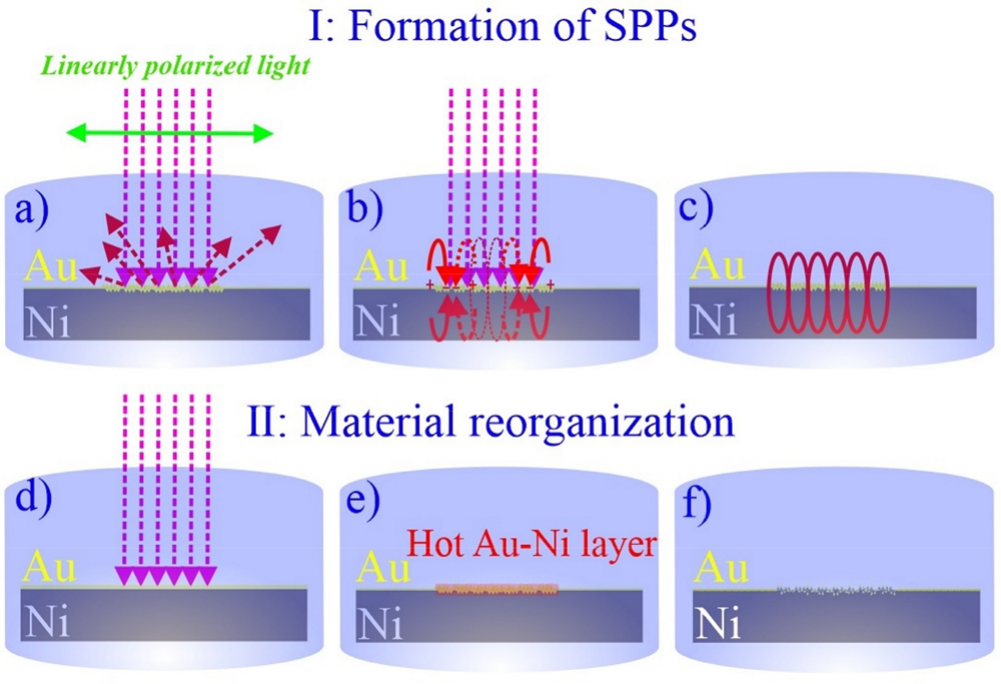
Schematic of mechanisms forming LIPSS. (I) Formation of surface plasmon polaritons (SPPs): (a) laser interaction with the surface and scattering on the roughened area; (b) formation of SPPs and interference between the incoming waves and SPPs; (c) LSFL generation induced by SPPs. (II) Material reorganization: (d) interaction of a femtosecond beam with Au-coated Ni; (e) laser softening of the Au/Ni surface and interface along with the initiation of hydrodynamic instability; (f) relaxation, surface rearrangement, and formation of LIPSS.

The formation mechanism of LIPSS by means of ultrashort pulses has not yet been fully explained due to the complexity of the process.^18,19^ A schematic illustrating the proposed mechanisms of LIPSS generation is shown in Figure 1. The interaction of matter with an ultrashort intense beam leads to self-organization of the surface and dynamic generation of nanoripples. In general, two concepts explain LIPSS formation: (I) the electromagnetic model and (II) material reorganization.^2^ The electromagnetic model is based on absorption and scattering throughout the irradiation time. The scattering of incident light at rough surface areas leads to surface excitation, oscillations of free electrons, and formation of surface plasmon polaritons (SPPs).^9^ Interference between all waves (incident, scattered, SPP)^20^ modifies the spatial distribution of the laser energy and thus forms LIPSS. The electromagnetic model has been widely accepted for LSFL formation.^9^

Matter reorganization takes place after the pulse and includes hydrodynamic instabilities, phase transitions, and the formation of microscopic defects that are responsible for LIPSS formation. Reif et al.^21^ proposed the instability to be analogous to hydrodynamic instabilities of molten layer films. In metals, ultrashort laser irradiation of the surface triggers the absorption of laser energy by surface electrons that leads to the formation of hot and excited electron gas while the lattice system stays cold, as explained by the two-temperature model.^22,23^ Since the femtosecond pulse is much shorter than the electron–lattice relaxation time (10^–10^ to 10^–12^ s),^24^ the heat transfer from electrons to the lattice by electron–phonon collisions occurs without any further heating of surface electrons. Thus, a soft liquid layer film with destabilized crystal structure can be formed after heat transfer from electrons^25^ to the lattice and finally surface roughening occurs upon heat dissipation. Surface relaxation and smoothing take place via competition between surface erosion and surface diffusion,^18^ generating self-organized micro/nanostructures. In the case of multipulse interaction with a Au-coated metallic surface, due to the coincidence of these effects and high possibility of SPPs formation, a combination of both mechanisms (Figure 1, I and II) can be expected. This may start with SPPs formation and end with material reorganization.

Up to now, the LIPSS formation on metals, alloys, semiconductors, and polymers has been mostly studied in air or vacuum, while only few reports exist regarding LIPSS generation in liquids,^26−43^ as laser patterning in liquids is more complex. Particularly, on some metals LIPSS generation cannot always be achieved in liquids, due to the physiochemical properties of the solid or the liquid characteristics. Studies frequently focused on varying laser parameters or changing the liquid thickness to obtain different periodicities. However, the surface topography or spatial periodicity were typically characterized without taking chemical composition, crystallinity, wettability, and deviation of ripples into account.

Mazur and co-workers^28^ observed the formation of smaller LIPSS periodicity on surfaces (Si, GaAs, GaP, InP, Cu, and Ti) in water, that correlated with the fast cooling of the molten surfaces in water during femtosecond laser irradiation and with nonlinear refractive index effects on the wavelength of the light. Also, Kobayashi et al.^39^ showed a correlation between LIPSS periodicity and liquid refractive index, with higher refractive index liquids leading to shorter periodicity.

Zhigilei and co-workers^44^ studied LIPSS on Cr in water experimentally and theoretically. They suggested a single-pulse femtosecond trigger phase explosion at the target’s surface, which transforms into a thin (∼40 nm) vaporized layer. The newly formed hot ablation plum transfers its temperature to water, and supercritical water can be formed. Simulation suggested the presence of Rayleigh-Taylor instability at the interface between the molten layer and supercritical water, trigger roughening, formation of morphological features, and self-organized structures. Sun and co-workers^34^ studied femtosecond multipulse LIPSS on an elastic alloy (48.35% Fe and 42% Ni) in water and ethanol, and it was proposed that increased water depth decreased the LIPSS periodicity. In ethanol, the concentration played a role, and the highest periodicity was observed for 80% ethanol. Moreover, at the highest liquid depth (2 mm), LIPSS transformed into conical features. Guo and co-workers^45^ presented the first study of femtosecond laser-induced surface colorization (purple to orange) of Cu in liquids, associated with nanoparticle-induced plasmonic absorption. Colorization of the Cu surface in liquid is lighter than in air and is related to smaller nanoparticles on the surface or a lack of hierarchical surface features that are responsible for light trapping.

In the work presented herein, multipulse femtosecond laser processing of Au-coated Ni foil in various liquids led to the formation of nanoripple structures with submicrometric spatial periodicity. Static contact angle measurements were performed to determine the surface wettability in comparison to pristine areas. In order to investigate the reason for decreased hydrophilicity, surface morphologies and chemical compositions were studied. Scanning electron microscopy (SEM) was applied to image the ripples including LSFL and HSFL and to study the periodicity created in ethanol, butanol, hexane, and water. Fast Fourier transforms were used to measure the LSFL and HSFL angular deviations from vertical orientation. The crystallinity of the Au-coated Ni surface in pristine and laser-treated zones was studied with grazing incidence two-dimensional micro X-ray diffraction (GIXRD). Confocal micro-Raman spectroscopy was carried out to examine the graphitization of surfaces laser-treated in ethanol, butanol, and hexane.

## 2 Materials and Methods

### 2.1 Laser Target

A polycrystalline nickel foil was obtained from Alfa Aesar (thickness of 0.5 cm and purity ≥99.9%). Gold was deposited on nickel by physical vapor deposition (PVD) of a Au wire (purity ∼99.95%). According to SEM imaging, the Au film had a thickness of about 75 nm. Solvents including ethanol, butanol, and hexane (p.a. 99.5%) were purchased from Sigma-Aldrich.

### 2.2 Femtosecond Laser Setup

**Figure 2:**
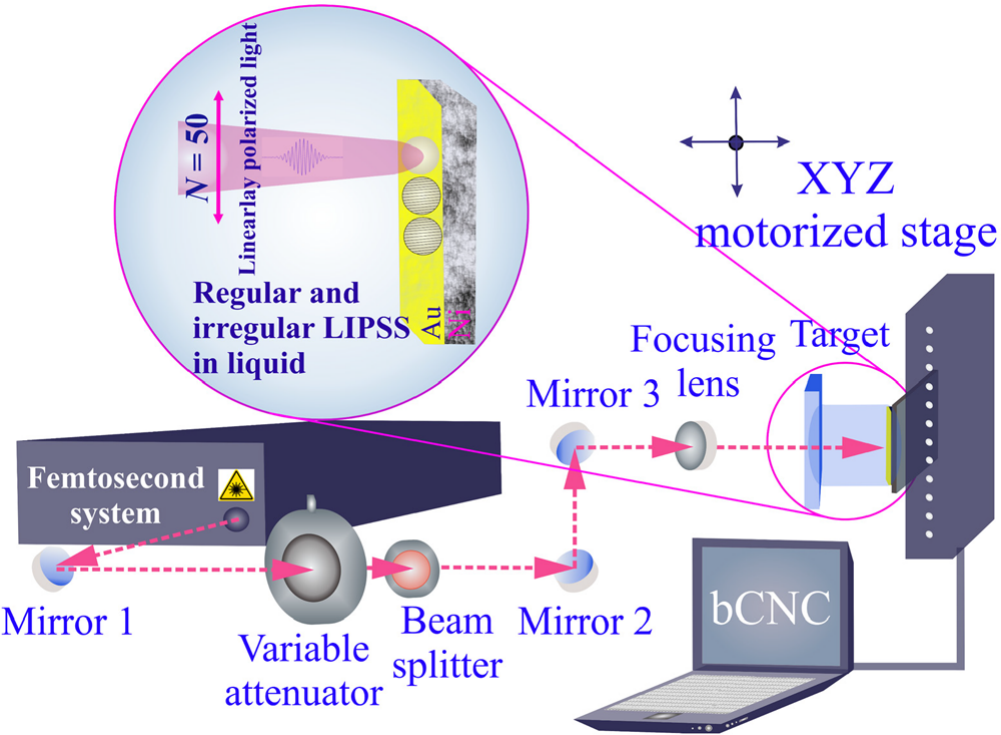
Schematic of the femtosecond laser setup for LIPSS generation on Au-coated Ni in liquids. LIPSS was created in ethanol, butanol, hexane, and water and included regular and irregular features, oriented perpendicularly to the linearly polarized beam (*N* = 50, 30 fs, 1 kHz, *E* = 100 μJ).

To produce periodic LIPSS by multipulse (*N* = 50) femtosecond laser processing of Au-coated Ni foil in various fluid media, a near-infrared amplified femtosecond laser (FEMTOPOWER compact PRO) at pulse duration (τ) of 30 fs, pulse repetition rate of (*f*_rep_) 1 kHz, wavelength (λ) of 800 nm and laser energy (*E*) of 100 μJ was used. A schematic of LIPSS generation in liquids is presented in Figure 2. The output radiation was linearly polarized with a zero angle of incidence (θ = 0). A variable attenuator including a beam splitter and polarizer (THORLABS) was used to modify the beam power and a power meter (Ophir Photonics) measured the beam intensity. The Gaussian beam was focused on the target by using a silver-coated parabolic mirror with a focal length *f* of 101.6 mm. A horizontally mounted cylindrical glass cell with a height of 15 mm was placed in the beam, with the sample mounted at the bottom of the cell capped by an optical window. Multipulse processing was performed by a motorized XYZ translational stage controlled by bCNC software.

### 2.3 Contact Angle Measurements

Static contact angle measurements of distilled water droplets (without changing the sample holder tilt) on pristine and femtosecond laser-induced ripples on a Au-coated Ni surface were carried out on a KRÜSS instrument (DSA100). The sample was cleaned with isopropanol and water in an ultrasonic bath for a few minutes and dried in an oven. After reaching room temperature, the sample was positioned on the manually operated stage, and a water droplet was deposited at the desired position. Then, the droplet was zoomed, focused, and photographed. The treated samples were positioned such that contact angles were measured perpendicular to the ripples. Video recordings revealed the droplet evaporation as a function of time. ImageJ software with a drop analysis plugin (designed by A. F. Stadler and D. Sage^46^) was used for the analysis of surface wettability. For better accuracy, a low bond axisymmetric drop shape analysis (LB-ADSA) model was applied which is related to applying the Young–Laplace fitting to the droplet image data. Moreover, this technique is able to precisely distinguish a drop’s baseline position. The software measures the right and left angles based on the shape of the droplet, obtaining a mean contact angle.

### 2.4 Scanning Electron Microscopy (SEM)

A FEI Quanta 250 FEG-SEM with an operating voltage of 0.2–30 kV, a Schottky field emission gun (beam current of ∼100 nA), and an Everhart-Thornley detector for secondary electrons were used to study the LIPSS morphologies and their orientation angle. For imaging, an operation voltage of 5 kV and a spot size of 2 μm were used. To process SEM images, the ImageJ software was used to measure periodicity and extract LIPSS profiles.

### 2.5 Grazing Incidence X-ray Diffraction (GIXRD)

An XRD system (Empyrean, PANALYTICAL; Cu–Kα radiation (1.54 Å), 45 kV and 40 mA) was used for phase and crystallinity analysis of femtosecond-generated LIPSS created on a metallic Au–Ni target in ethanol, butanol, hexane, and water. The Z-height adjustment was done for each sample positioned on a remote-controlled XYZ stage and observed by a charge-coupled device (CCD) camera. A micronozzle with a diameter of 300 μm was used to obtain a rectangular slim beam with a length of 6 mm. In order to reduce noise and avoid defocusing, a parallel beam X-ray collimator was mounted. To obtain higher resolution and surface sensitivity, the angle of incidence (ω) was fixed at 6°. Two-dimensional (2D) micro-XRD was achieved via grazing-incidence diffraction (GID). The 2D-detector position (GaliPIX^3D^, Malvern PANalytical) was changed from 20° to 100°.

### 2.6 Confocal Micro-Raman Spectroscopy

Micro-Raman scattering analysis was done on a Horiba XploRATM INV at room temperature equipped with a thermo-electrically cooled charge-coupled device (CCD) detector and a fully *XY* motorized stage. A diode laser at a wavelength of 532 nm was used as an excitation source. The maximum power of the laser was 100 mW and tunable by a filter to decrease intensity. The Raman system was connected to an optical microscope (Nikon Eclipse TiU) to locate the desired area and focus the laser beam. Before starting the measurements, the micro-Raman spectrometer was calibrated. To focus the beam on the laser-treated areas an objective of 10x was used. Micro-Raman results of LIPSS on Au-coated Ni foil were recorded between 300 to 3000 cm^–1^. A filter was used to acquire 50% of the total beam intensity. To achieve higher resolution, a holographic grating of 1200 grooves/mm was applied. Micro-Raman spectroscopy was done at 1 s acquisition time and for each spectrum, 30 measurements were accumulated. HORIBA Scientific’s LapSpec 6 spectroscopy suite software was used to collect the spectra and subtract the background. Signal processing and smoothing of peaks were done by the method of Adjacent-Averaging and a window size of 6 with polynomial order of 2.

## 3 Results and Discussion

In the following, the preparation of different laser-processed surfaces will be described first, followed by an evaluation of their wettability by water, a common test for LIPSS. To better understand the correlation between structure and function, the LIPSS will then be characterized by SEM, GIXRD, and confocal micro-Raman spectroscopy.

### 3.1 Laser Processing

Periodic surface nanostructures were formed using a linearly polarized femtosecond laser system (800 nm, 1 kHz, *E* = 100 μJ) by applying 50 pulses on a Au-coated Ni surface, either in ethanol, butanol, hexane, or water. The pulse number and laser energy were kept constant in order to investigate the role of the fluid on the contact angle, chemical composition, morphology, periodicity, orientation, and deviation angle of the nanoripples.

### 3.2 Wettability Analysis

**Figure 3:**
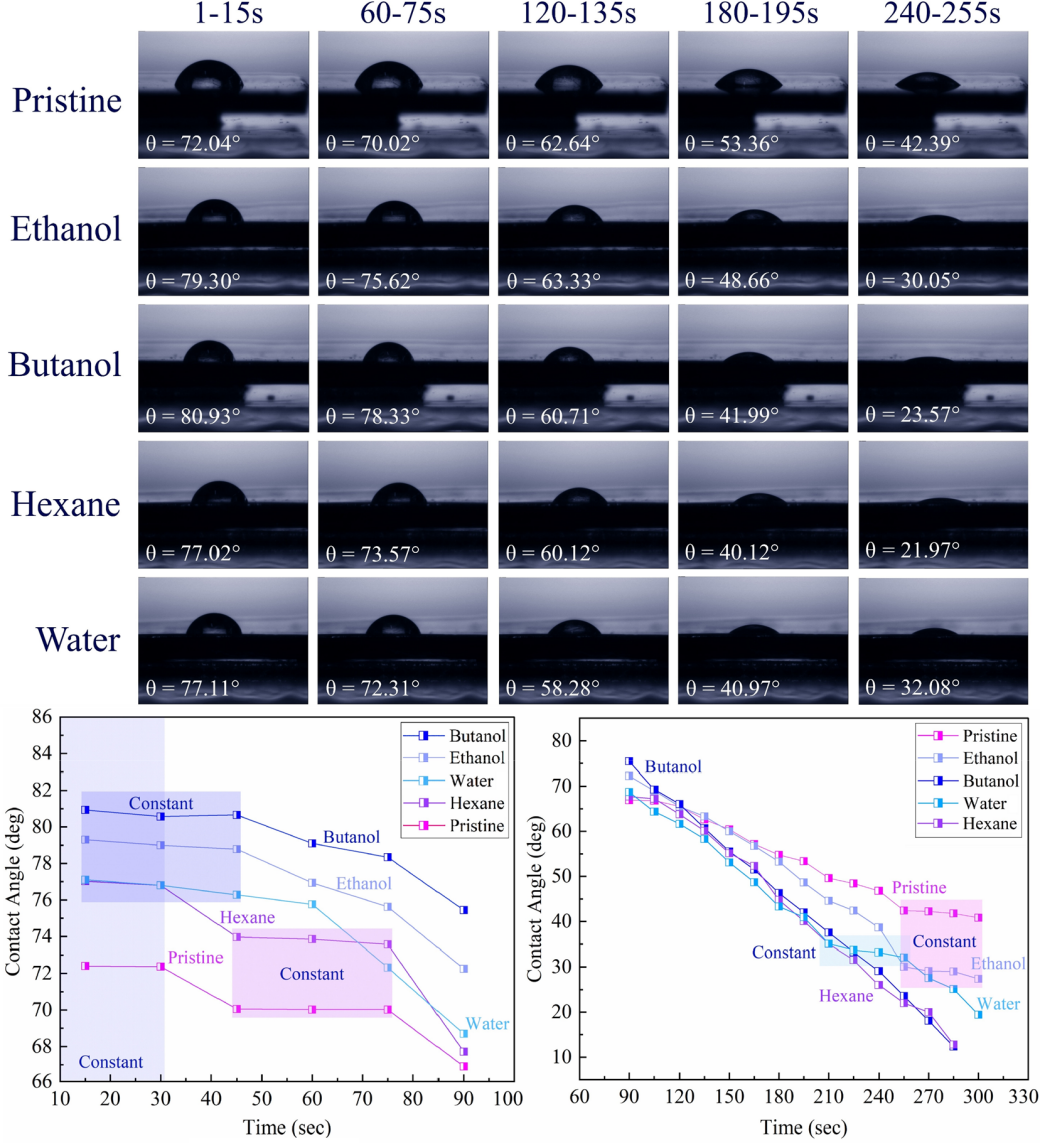
Representative analysis of the water contact angle on a Au-coated Ni surface, both for pristine and areas femtosecond laser-treated in various fluids (*N* = 50, 1 kHz, *E* = 100 μJ). Typical images in specific time windows were chosen to display the contact angle changes. The ripple direction is approximately perpendicular to the image plane.

To study the surface wettability by water, of pristine areas with regions laser-treated in various fluids, contact angle measurements were made at room temperature. To create a plot of contact angle as a function of time, the droplet evaporation was recorded as well. Selected images of droplets at specific time intervals are shown in Figure 3. The contact angle remained constant for about 30 s for all and was constant for 45 s for butanol, ethanol, and water treated areas (Figure S1). Due to continued water evaporation, for butanol and hexane, the contact angle went through a linear decrease from 90 to 300 s, while for pristine and water areas a linear decrease occurred from 90 to 210 s; for ethanol it occurred from 90 to 255 s. For water and ethanol, there is another stage from 210 to 255 s with evaporation occurring while the contact angle remains constant. Moreover, only for pristine and ethanol areas did the last stage of evaporation occur via receding of the contact line at the solid/liquid interface instead of changing the contact angle (225–300 s).

For all laser-treated areas, the *initial* contact angle is higher than that on a pristine area, which is a result of the formation of ripples^47^ and surface roughening^48^ and also due to surface chemical modifications.^49^ Typically, surfaces with θ > 90° are hydrophobic and below 90° are hydrophilic.^50^ Since all the measured angles are below 90°, the surface of nontreated and treated areas both showed hydrophilic tendencies. However, especially in butanol, the contact angle increased to 80.9°, confirming that laser-treated surfaces tend to be in a hydrophobic status, which is not only due to roughness and reduced effective surface tension,^51^ but also due to chemical composition (see below). The relation between ripple direction and contact angle may also be of interest, as contact angles may vary in directions parallel and perpendicular to the ripples,^52^ or even exhibit hydrophilic and hydrophobic behavior parallel and perpendicular to the ripple direction, correspondingly.^53^ Herein, contact angles were measured perpendicular to the ripple direction, but due to varying deviations in the ripple direction, a direct relationship between ripple direction and contact angle could not be determined.

The observed maximum decrease of wettability after treatment in butanol motivated further surface characterization by electron microscopy and X-ray diffraction to relate surface properties with surface structure/composition. Confocal Raman spectroscopy is a powerful technique to identify carbonaceous compounds (e.g., graphitization or amorphization) on surfaces laser-processed in various fluids, which may modify wettability.

Typically, graphite shows a hydrophobic nature which may be further affected by hydrocarbon contamination from air pollutants.^54^ Compared to the pristine surface, the formation of imperfect graphite and hydrocarbons on the periodic surface structures, and graphite amorphization by continuous multipulse femtosecond laser processing, may thus contribute to the increase in contact angle.

### 3.3 Surface Morphology Studies

**Figure 4:**
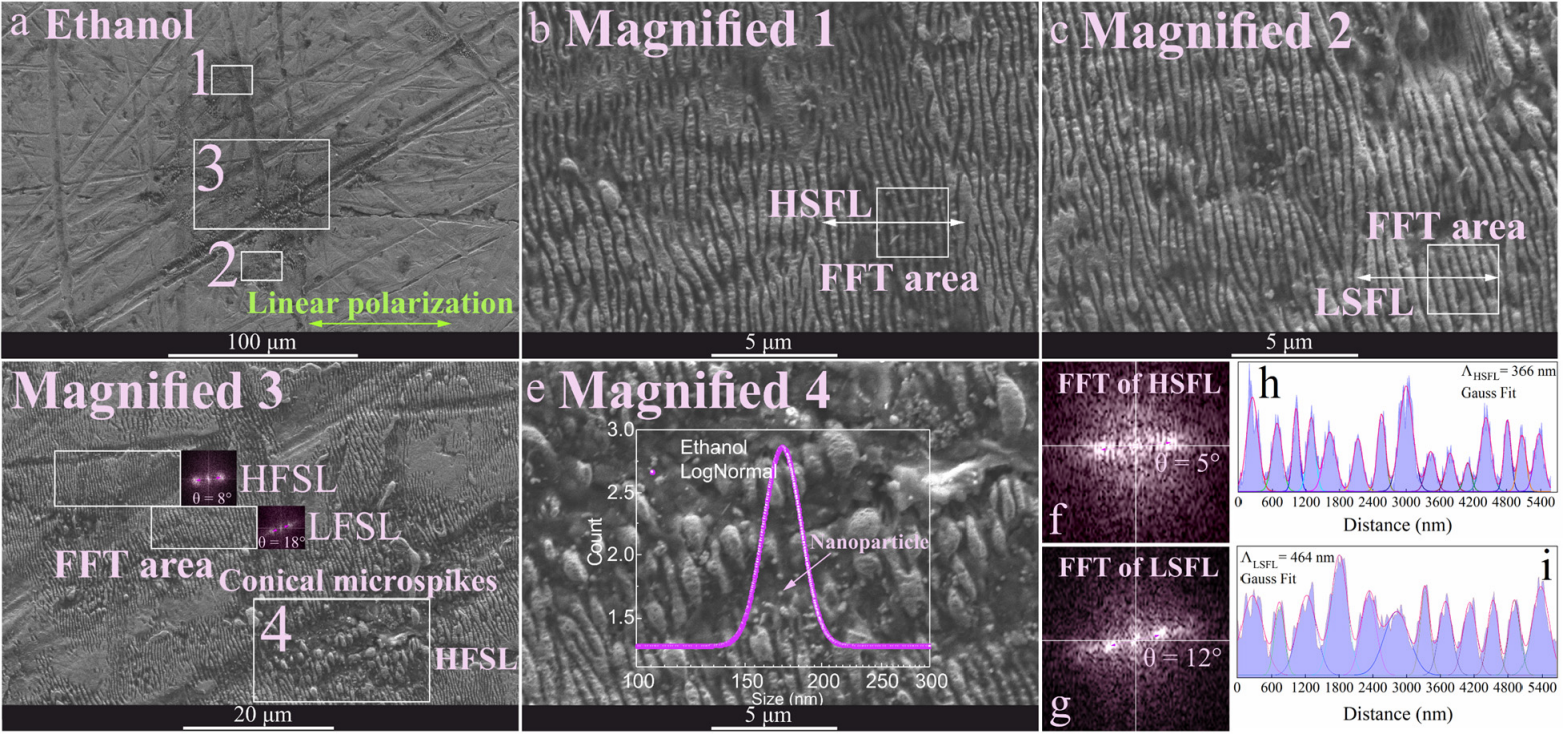
(a) Overview SEM image of surface morphologies of Au-coated Ni surface femtosecond laser treated in ethanol (*N* = 50, *E* = 100 μJ), including (b,c,d,e) selected higher magnification images. (b,c,d) The magnified areas show LIPSS, (d) conical microspikes, and (e) trapped nanoparticles. (d,f,g) FFT patterns related to HSFL and LSFL show the deviation angle of self-organized subwavelength features with respect to the horizontal beam polarization. (h,i) Periodicity profiles for HSFL and LSFL with their corresponding accumulative Gauss fit based on magnified SEM images 1 and 2.

**Figure 5:**
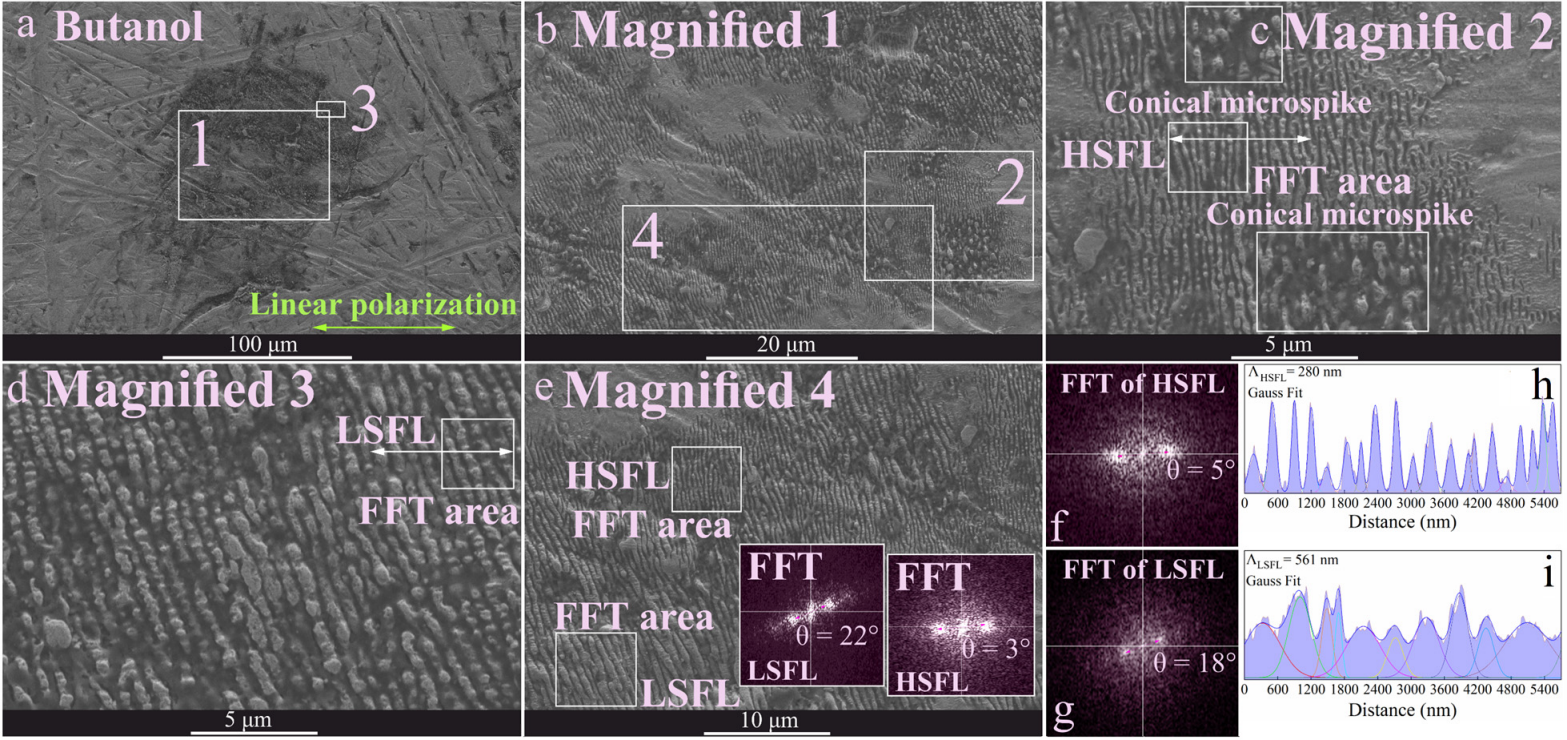
(a) Overview SEM image of surface nano/microstructures of Au-coated Ni surface femtosecond laser processed in butanol (*N* = 50, *E* = 100 μJ): (b,c,d,e) selected higher magnification images, (e,f,g) FFT patterns, and (h,i) periodicity profiles for LSFL and HSFL. Accumulative Gauss fit was applied for periodicity profiles extracted from magnified SEM images 2 and 3.

**Figure 6:**
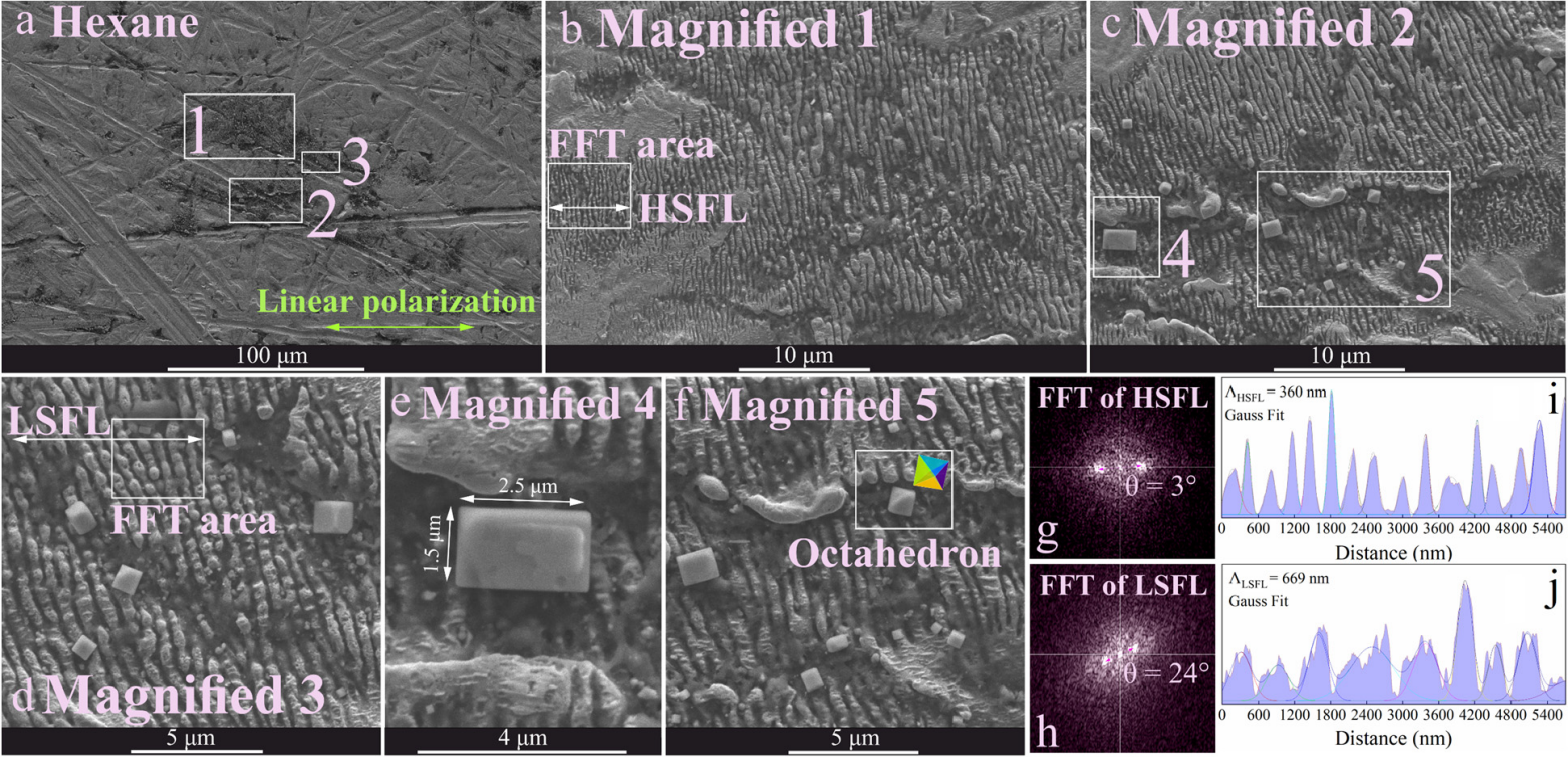
(a) Overview SEM image of LIPSS morphologies of Au-coated Ni surface femtosecond laser treated in hexane (*N* = 50, *E* = 100 μJ), and (b,c,d,e,f) corresponding higher magnification images. Magnified areas of SEM images display LIPSS together with trapped cubic particles. (g,h) FFT patterns (deviation angle study) and (i,j) periodicity profiles of HSFL and LSFL with their corresponding accumulative Gauss Fit taken from magnified images 1 and 3.

**Figure 7:**
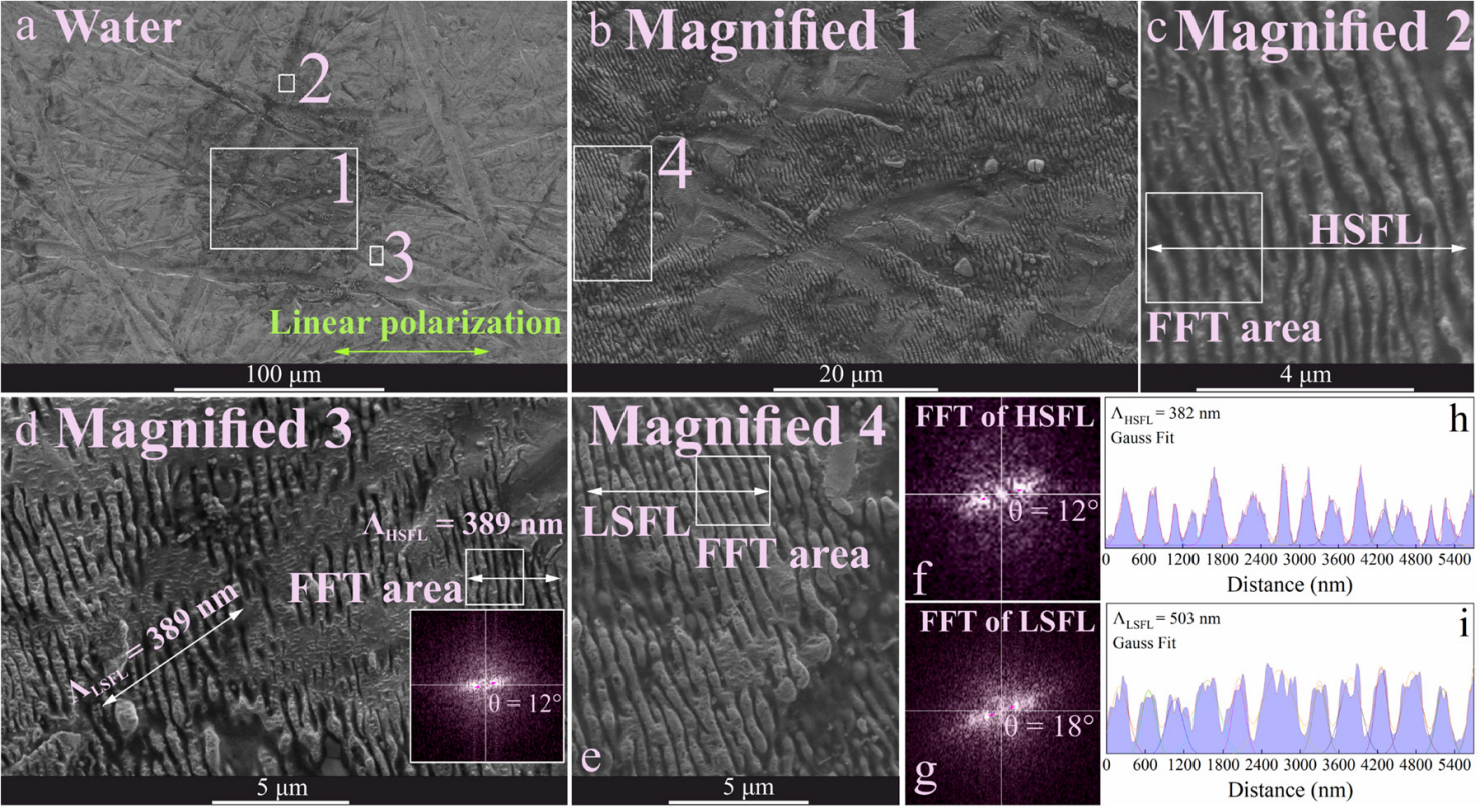
(a) Overview SEM image of subwavelength LIPSS of Au-coated Ni surface femtosecond laser treated in water (*N* = 50, *E* = 100 μJ), including (b,c,d,e) corresponding higher magnification images. (f,g) FFT patterns and (h,i) periodicity profiles of HSFL and LSFL with their corresponding accumulative Gauss Fit were from specified areas on magnified images 2 and 4.

SEM images are presented in Figures 4 to 7, displaying the topography and morphology of self-organized subwavelength ripples produced by multishot femtosecond laser processing of Au-coated Ni in ethanol, butanol, hexane, and water, respectively. A liquid thickness of 12 mm was used in all cases; thus the initial pulse duration (30 fs) broadened due to the pulse traveling from air/glass to liquid. Nevertheless, the final pulse duration (Table S1) did not exceed the femtosecond range.^55,56^

The orientation of LIPSS on Au-coated Ni is typically perpendicular to the horizontal beam polarization. In previous work with the same femtosecond system, this orientation was also observed on steel.^57^ Furthermore, several metals responded the same way to the beam polarization as the presented work.^28,58−63^ Even though a horizontally polarized beam was applied by the used femtosecond setup, the final orientation of LIPSS in all liquids was frequently tilted anticlockwise by angles of 3° to 24° (see below). A similar change in LIPSS orientation, again with the original beam linearly polarized in the horizontal direction, was also observed for Cu and semiconducting materials femtosecond processed (*N* = 200) in water.^28^ Also, a shift in the spatial periodicity angle of LIPSS was presented by Zhang and Sugioka^38^ for processing Si in water via crater ablation (*N* = 400, 500, 800, 1200, 1500, and 2000). Typically, laser processing in liquids is paralleled by liquid turbulence^45^ at the liquid/solid interface which may account for changes in the ripple direction. Moreover, laser processing in liquids can modify the focal length,^64^ which mostly depends on the refractive index and thickness of the fluids. Thus, refraction can not only change the spot diameter, but may also affect the ripple periodicities. Such modifications in various liquid media are collected in Table S2. The largest change in focal length occurred in butanol due to its refractive index being higher than that of the other liquids.

However, the extent of deviation depended on the type of self-organized structures. In order to precisely study the deviation angle, fast Fourier transform (FFT) was applied to selected images of specified areas. The highest deviation was observed for LSFL in hexane (Table 1). In contrast, HSFL were mostly close to vertical, with the lowest angle deviation observed for hexane (3°), but the highest deviation for water (Figure 7) at 12°. In ethanol, butanol, and hexane the deviation angle difference between LSFL and HFLS was higher than in water.

**Table 1:** Nanoripple Periodicities and Their Deviation Angle for a Au-Coated Ni Surface Femtosecond Laser-Processed in Various Fluids

Fluid	LSFL/nm	θ_LSFL_	HSFL/nm	θ_HSFL_
ethanol	464	12	366	6
butanol	561	18	280	5
hexane	669	24	360	3
water	503	18	382	12

HSFL was frequently present for butanol (Figure 5) with the lowest periodicity of 280 nm. Presumably, the refraction and reflection of the beam at the bubble wall are responsible for HSFL features.^15^ The largest periodicity for LSFL (669 nm) was measured for hexane (Figure 5). Moreover, in some areas, LSFL split to HSFL, which may be due to surface defects that trigger the division of ripples. In addition to the self-organized structures, there were some microspikes where nanoparticles were trapped. For ethanol (Figure 4) a median size of 173 nm was measured for spherical nanoparticles. A small difference in laser energy deposition on the treated area due to beam scattering or refraction related to the produced nanoparticles, solvent photolysis or byproduct formation, may affect the surface morphologies. Thus, these locations absorb slightly more energy, so that ripples can transform into grooves and microspikes.

In a simple view, laser ablation initiates with a slight color change of the material due to a change in chemical composition; later on, defects can be generated and self-organized surface structures are formed via material reorganization. The plasma temperature upon femtosecond pulses is 5000–7000 K^65^ with a cooling rate of molten droplets around 10^12^ K s^–1^.^66^ For hexane, cubic and octahedral Au particles were detected while faceted particles generally tend to spherical shape for surface energy minimization. Also, atomistic modeling confirmed the existence of different shapes for Au nanoparticles considering their thermal stabilities.^67^ In fact, these trapped cubic nanoparticles are frozen molten droplets ejected from the surface during phase explosion^56^ and material reorganization. Then, as a result of fast cooling processes, these cubic and octahedral shapes are well preserved.

Kabashin and co-workers^68^ studied the mechanism of ultrashort laser ablation for metal nanoparticle generation. It was suggested that the effective laser energy penetration depth is about ∼250 nm. Thus, an increase in electron–phonon interaction and fast electron–phonon relaxation inside the confined area may deliver sufficient energy to initiate a spallation process with large molten droplets ejected.

Also, the large Au cuboid observed on the surface (Figure 6e) is much larger than the LIPSS periodicity. This may be related to the direct effect of laser light on the Au surface apart from material reorganization (indirect process) and results from surface disintegration. This occurs upon inhomogeneous beam deposition on the surface, as absorptivity of the laser light increases on a certain area due to carbonization. Since cuboid Au particles were only detected for hexane, whereas small spherical particles were formed in ethanol (Figure 4), it is apparent that the fluid characteristics can potentially control the particle morphologies.

Some HSFL was also formed in water (Figure 7), far from the center and closer to the periphery of the laser-treated area. Also, the HSFL deviation angle is greater than 10°, in contrast to other liquids where it is close to the deviation angle for LSFL. Furthermore, ripple features were mostly short and noncontinuous.

As mentioned before, most of the LIPSS generation studies reported in the literature were carried out in air to generate well-controlled ordered LIPSS, but generating nano/microstructures in liquids may be of importance. Nevertheless, several factors should be considered for high-precision processing of LIPSS in liquids. For instance, an advanced cell design would be needed to continuously exchange the liquid with a fresh one, so that micro/nanoparticles ejected from the surface cannot trigger beam scattering and subsequent inhomogeneous energy deposition on the processed surface. Additionally, water can be a proper option to avoid carbon contamination since surface carbonization may also affect laser absorptivity. Also, it is advantageous to polish the surface before laser processing or use a single pulse together with an improved cell with a very thin liquid layer to minimize defect generation.

Only few studies exist regarding LIPSS formation on metals/alloys in liquids and an overview is presented in Table 2. The orientation of LIPSS was mostly perpendicular to the linear polarization of light but rarely parallel. In two previous studies of LIPSS generation on stainless steel^39^ and Zn^40^ LIPSS formation was not achieved in ethanol. The deviation angle of LIPSS orientations on metals in liquids, their surface chemical composition, crystallinity and wettability have mostly not been considered though.

**Table 2:** Overview of LIPSS Generation on Metals/Alloys in Liquids by Short-Pulsed Lasers

metal/alloy	liquid	laser parameters	periodicities/nm	polarization	LIPSS deviation from polarization direction	LIPSS direction to the polarization	characterization techniques	ref
Au-coated Ni	1. Ethanol	Femtosecond laser	LSFL (464, 561, 669, 503) and HSLF (366, 280, 360, 382) produced in all liquids.	Linear	Studied by FFT. Occurred in all liquids and all types of LIPSS	⊥	1. Optical microscopy	Presented work
	2. Butanol	800 nm, 1 kHz, 30 fs, *N*50					2. SEM/FFT	
	3. Hexane	100 μJ					3. μGIXRD (2D,1D)	
	4. Water						4. Micro-Raman spectroscopy	
							5. Contact angle	
Cu	Water	Femtosecond laser	HSFL (280) for Cu and HSFL (200) for Ti	Linear	Not studied but relatively high deviation is visible in water	⊥	1. SEM	(28)
Ti		800 nm, 1 kHz, 100 fs, *N*_200_						
		2.5 J cm^–2^						
Cr	1. Water	Femtosecond laser	HSFL in all liquids.	Linear	Not studied but deviation is slightly visible in water	∥ and ⊥	1. SEM	(30)
Ti	2. Ethanol	775 and 387 nm, 2 kHz, 200 fs, ∼ 0.11 J cm^2^	[775 nm]: water (80–120), ethanol (120–180), chloroform (150–200)				2. Atomic force microscopy (AFM)	
W	3. chloroform		[387 nm]: water: (75–120), ethanol (60–90), Chloroform (150–200)					
Constant elastic alloy	1. Water	Femtosecond laser	LSFL and HSFL (676 to 383) in water. HSFL (260 to 380) in ethanol	Linear	Not studied but deviation is visible in water and ethanol	⊥	SEM	(34)
	2. Ethanol	800 nm, 1 kHz, 120 fs, *N*200						
		0.58 J cm^2^						
W	Ethanol	Femtosecond laser	HSFL for W (320 to 340) and LSFL for Mo (240 to 520)	Linear	Not studied but deviation is visible in SEM images	⊥	1. SEM	(35)
Mo		800 nm, 1 kHz, 30 fs, *N*1000					2. Confocal microscopy	
		50 to 250 μJ						
Cu	1. Water	Fiber femtosecond laser	Chaotic irregular LIPSS are visible in SEM.	Linear	Not studied but deviation is visible in SEM images	∥ and ⊥	SEM	(36)
	2. Ethanol	1030 nm, 1 kHz	LSFL (620) in water, LSFL (575) in ethanol and LSFL (630) in methanol					
	3. Methanol	1.5 ps, *N*100 and *N*1000						
		0.36 and 0.9 J cm^–2^						
Stainless steel	1. Ethanol	Picosecond laser	No LIPSS in ethanol and decane.	Linear	Not studied but deviation is slightly visible in water	⊥	SEM	(39)
	2. Water	1030 nm, 50 ps, 10 kHz	LSFL (526) in water and HSFL (482) in Sugar syrup					
	3. Sugar Syrup	0.15 to 0.76 J cm^–2^						
	4. Decane							
Zn	Ethanol	Femtosecond laser	No LIPSS in ethanol	Linear	-	-	1. Optical microscopy	(40)
		800 nm, 1 kHz, 30 fs, *N*1000	However, some broken ripples were presented.				2. SEM	
		2.5 J cm^–2^					3. XRD	
							4. Fourier transform infrared spectroscopy (FTIR)	
							5. Hardness	
Ti, V, Nb, Ta, Mo, W, Fe, Pd, Pt, Ni, Au, Ag, Cu, and CuZn	Acetone	Femtosecond laser	UHSFL/LSFL were formed for group IVB–VIB transition metals.	Linear	-	Marangoni bursting was detected.	1. SEM	(42)
		1045 nm, 457 fs, 100 kHz, 6 μJ	LSFL and hole-rich microstructures were generated for group VIII and IB/IB–IIB.			UHSFL where ⊥ to the curvatures of LSFL	2. Raman spectroscopy	
							3. XPS spectroscopy	
							4. FTIR spectroscopy	

During the femtosecond beam interaction with the surface, liquid evaporation can initiate Marangoni bursting and consequently molten layer disintegration.^42^ Presumably, the fluid dynamics (e.g., vortexes and flows) aside from the beam polarization may be a significant factor to govern the formation of irregular structures including circular or crisscross LIPSS.^43^

### 3.4 Micro-GIXRD Analysis

**Figure 8:**
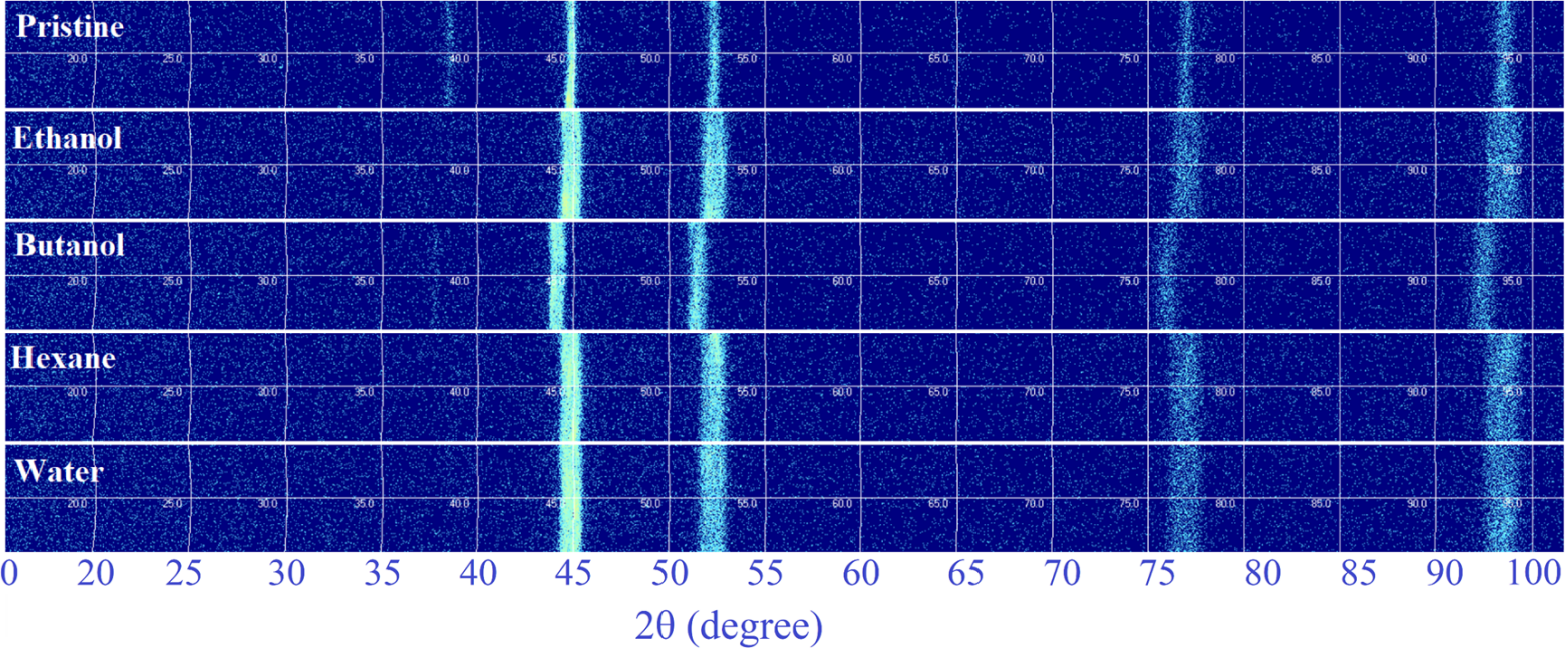
2D-μGIXRD analysis of Au-coated Ni, pristine and femtosecond laser processed (*N* = 50, 1 kHz, *E* = 100 μJ) in various fluids.

The 2D and 1D micro-GIXRD patterns of LIPSS created by femtosecond laser pulses on Au-coated Ni are shown in Figure 8 and Figure S2. Polycrystallinity is also confirmed by the sections of diffraction rings in 2D micro-GIXRD, as the crystallites were randomly oriented. Since the microbeam is focused on these surface nanostructures, the lines are not continuous but “spotty” which can be related to dissimilar crystallite sizes, since the periodicities on each laser-treated zone in various fluids were also different. Thus, the 2D pattern consisted of isolated reflection spots and corresponds to coarse-grained phases.^69^

Also, a narrower pattern is seen in pristine areas, which can be related to defect structures (e.g., holes) or microspikes on the laser-treated zones. However, it is typically related to the nanoripples which act as nanograting that may broaden the diffraction peaks and transform the sharp top to flattened coarse ones. Based on the Scherrer theory,^70^ peak width (ß) and crystallite size (*L*) are inversely related. Thus, the peak widening can partially relate to the crystallite size, which was analyzed by Williamson-Hall (WH) plots of samples treated in various fluids (Table 1).

**Figure 9:**
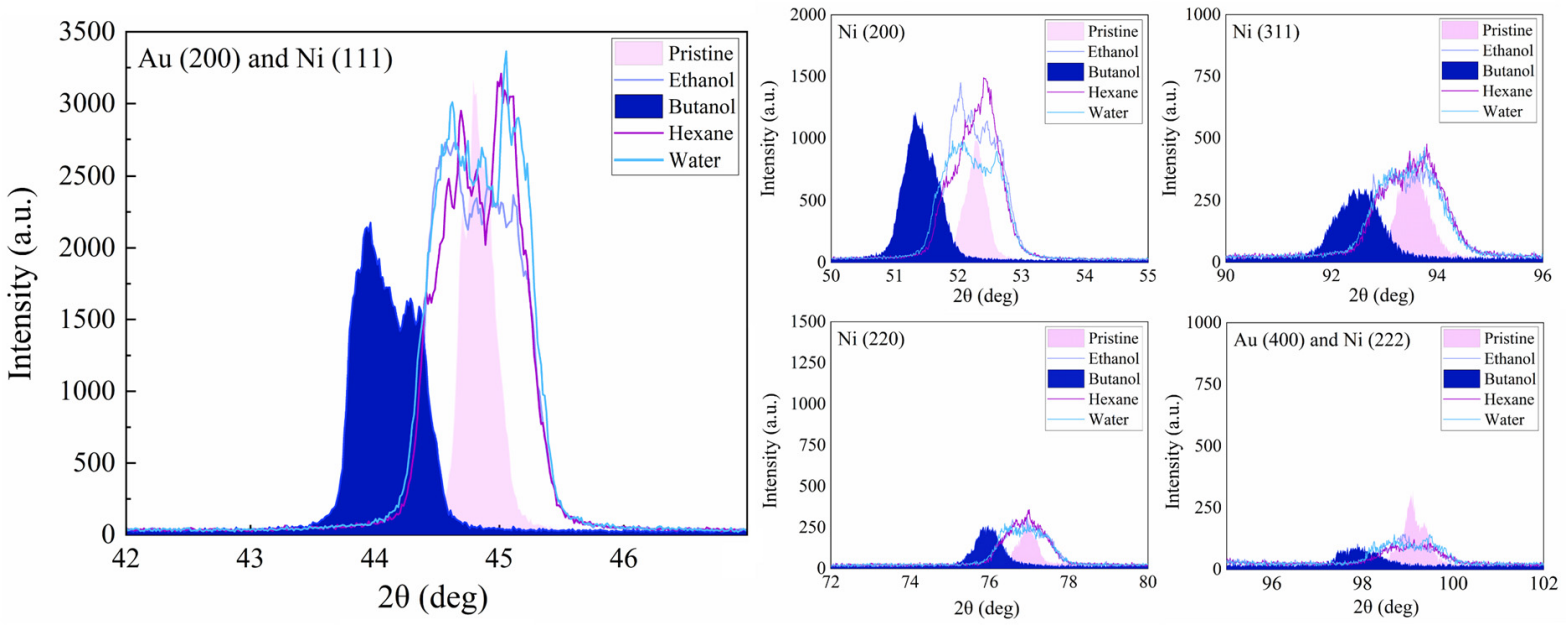
Magnified regions of 1D-μGIXRD on Au-coated Ni, pristine and femtosecond laser treated in various liquids, displaying Au (200), Ni (111), Ni (200), Ni (311), Ni (220), Au (400), and Ni (222).

Figure S2 displays Ni peaks (44.73°, 52.13°, and 76.84°; to JCPDS card no. 01-071-4655), for the pristine target and for regions after processing in ethanol, hexane, and water. Figure 9 shows magnified zones of the 1D-μGIXRD results in Figure S2. For areas treated in butanol (Figure 9), Ni (111), (200), and (220) peaks were shifted toward lower angles of 44.11°, 51.39°, and 75.92°, rather matching JCPDS card no. 01-071-4653, that has a larger cell volume and thus a greater *d-*spacing between atomic planes (44.28°, 51.59°, and 75.97°). This slight shift is analogous to the work of Assefa et al.^71^ on gold where a thin film was excited by a femtosecond laser and thermal expansion was proposed as reason.

**Figure 10:**
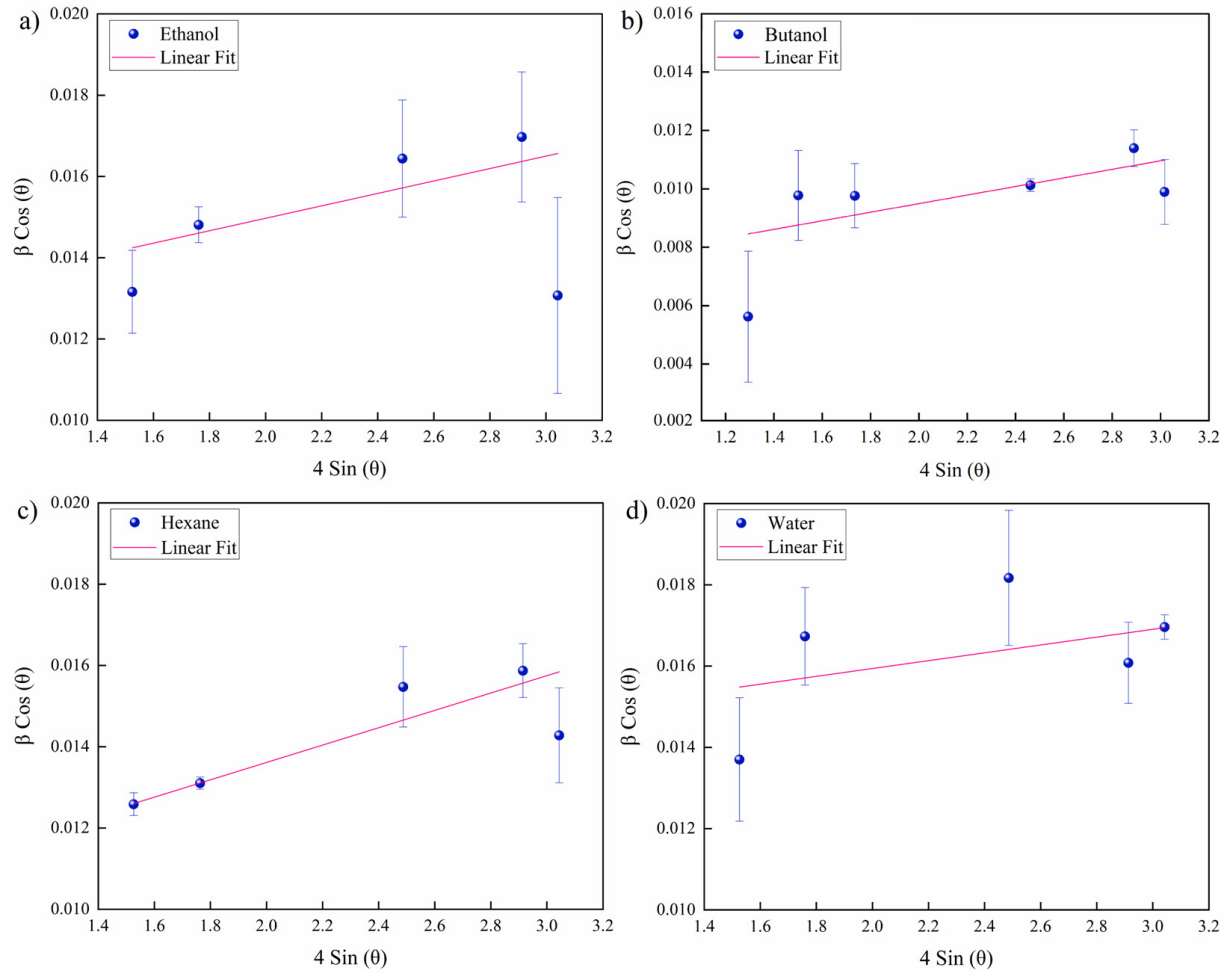
Microstrain and crystallite size analysis by Williamson–Hall plots related to LIPSS on Au-coated Ni, femtosecond laser processed in fluids (*N* = 50, 1 kHz, *E* = 100 μJ). The actual values are compared to predicted values to measure the root-mean-square error (RMSE) for the linear fit: (a) ethanol, RMSE = 0.001; (b) butanol, RMSE = 0.001; (c) hexane, RMSE = 0.0007; (d) water, RMSE = 0.001.

The microstrain (ε) and crystallite size (*l*_WH_) were assessed for areas laser-processed in various fluids (Figure 10) via Williamson–Hall plots^72^ by attributing the slope and inverse intercept of the linear fit to ε and *l*_WH_, correspondingly. Table 3 shows results of the evaluated WH plots. The larger lattice deformation (expansion) in butanol seems dominated by tensile stresses due to thermal expansion, an increase of cell volume and *d*-spacing rather than compressive forces (smaller *d*-spacing). Furthermore, all strain values resulted in positive slopes, thus lattice compression was not observed. The smallest crystallite size of ∼10 nm was observed for water. A similar size was measured for ethanol, while it was largest for butanol (∼21 nm). For hexane, the crystallite size was intermediate, but the microstrain was highest. Since the largest self-organized structures were detected for hexane (Figure 6j; Table 1) along with several small and large nanoparticles (Figure 5f), the pronounced fs laser-induced phase explosion^56^ may thus trigger a higher accumulation of deformation zones during consecutive pulses.

**Table 3:** Williamson-Hall Plots Analysis of LIPSS on Au-Coated Ni Produced by Femtosecond Laser-Treating Surfaces in Ethanol, Butanol, Hexane, and Water (*N* = 50, 1 kHz, *E* = 100 μJ)

fluid	slope	microstrain	intercept	crystallite size (nm)
ethanol	0.0015	1.53 × 10^–3^	0.011	11.7
butanol	0.0014	1.47 × 10^–3^	0.006	21.2
hexane	0.0020	2.14 × 10^–3^	0.009	14.9
water	0.0009	0.97 × 10^–3^	0.014	9.9

### 3.5 Analysis of Carbonaceous Compounds

**Figure 11:**
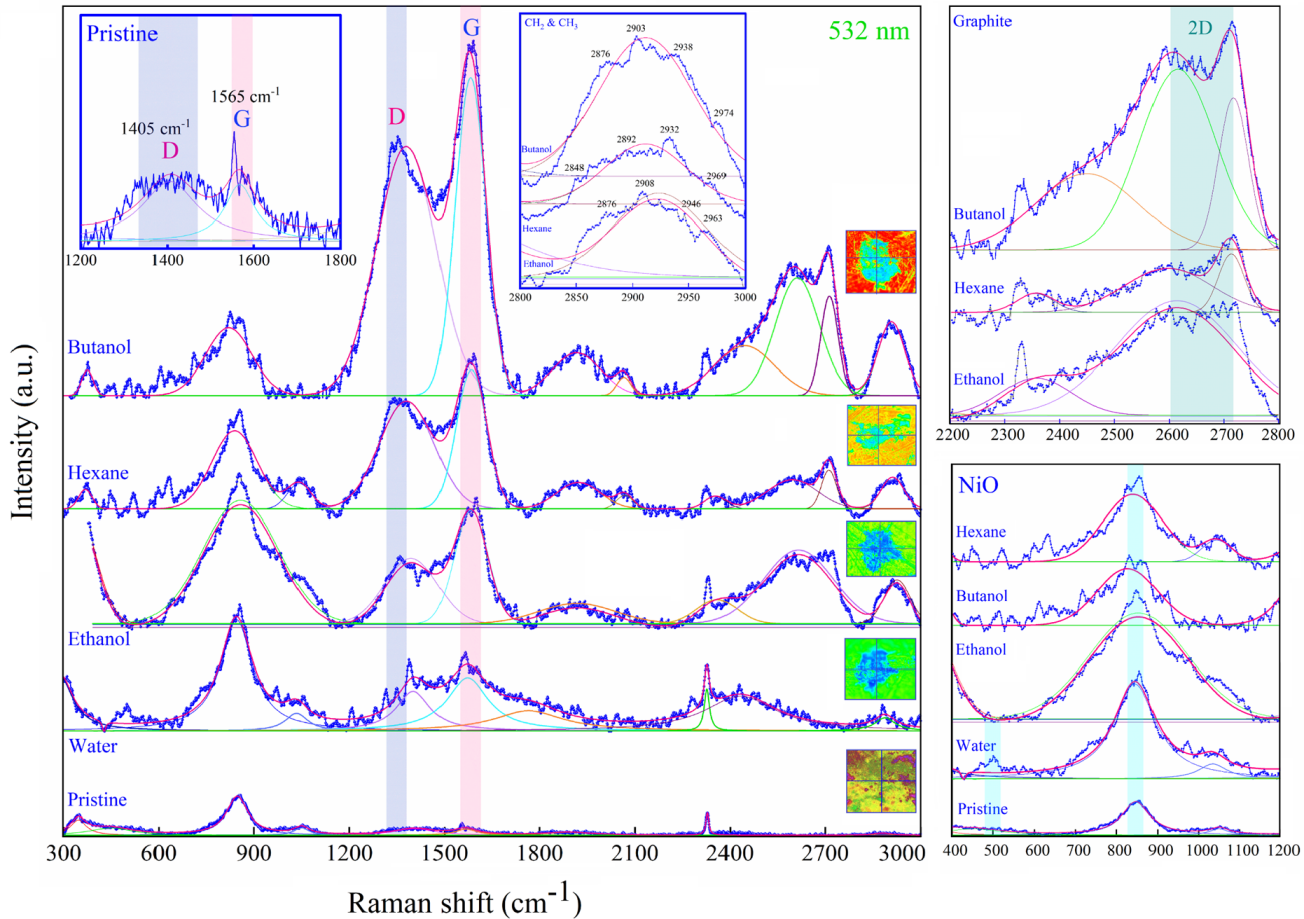
Micro-Raman spectroscopy analysis of Au-coated Ni for pristine areas and femtosecond laser-treated zones in various liquids (*N* = 50, 1 kHz, *E* = 100 μJ). Due to the strongly varying peak intensities, the studied liquids are ordered differently than in the other figures. Cumulative fits of Gauss and Lorentz (pink solid line) and respective deconvoluted peaks are selected based on the best *R*^2^. Pristine (Lorentz fit, *R*^2^: 0.95); water (Lorentz fit, *R*^2^: 0.93); hexane (Gauss fit, *R*^2^: 0.96); ethanol (Gauss fit, *R*^2^: 0.93) and butanol (Gauss, *R*^2^: 0.98). Magnified areas correspond to signature peaks of CH_2_ and CH_3_, graphite (D, G, and 2D-band) and NiO. 3D reconstruction of optical images shows the position of micro-Raman data acquisition.

Confocal micro-Raman spectra of Au-coated Ni, both for pristine areas and LIPSS for various fluids, were recorded between 300 to 3000 cm^–1^ at a laser excitation wavelength of 532 nm (Figure 11). In the Raman analysis, an average of ten different femtosecond treated zones and pristine areas were studied each, as presented in Figure S3. For ethanol (1393 cm^–1^, 1582 cm^–1^), butanol (1383 cm^–1^, 1585 cm^–1^), and hexane (1381 cm^–1^, 1584 cm^–1^) a broad double peak was detected, absent for pristine and water areas. These peaks correspond to the formation of graphitic carbon^73^ on femtosecond laser-treated areas which include D (defect) and G (graphite) bands. For extended Raman analysis, an average of ten different femtosecond treated zones and pristine areas were studied each, as presented in Figure S3.

Generally, long-pulse and short-pulse laser interaction with organic fluids (organic precursors) triggers solvent pyrolysis and photolysis, respectively,^5,6,74,75^ so that carbon byproducts can occur. In higher carbon-content fluids like hexane and toluene, laser pyrolysis/photolysis triggers the formation of graphite networks or amorphous carbon remaining in the fluid;^6,74^ thus carbon deposition on the laser-treated areas in hexane is lower than in butanol. In previous studies, multishot laser processing of Ni in butanol showed an increase in incubation behavior and an increase in carbon deposition in the craters.^76,77^ The amount of carbon deposition can affect the total intensity of the D-line and G-line.

The defect ratio of the D-band to G-band (*I*_D/G_) for ethanol, butanol, and hexane are 0.82, 0.80, and 0.85, respectively. To measure the *I*_D/G_, the average intensities were considered as presented in Table 4. The ethanol area contains the lowest intensity for its D band and G band (Table 4) among carbon-containing solvents, and the highest intensity of the G peak was found in butanol. More graphite carbon was observed for butanol and hexane, but in hexane a higher number of defects was present in the deposited graphite layer. Also, the width of the D-band was larger than that of the G-band, which is related to structure perturbation (e.g., exfoliation) in graphitic carbon. 2D-band (two phonon lattice vibration)^78^ peaks were observed for graphitic carbon in areas femtosecond laser-treated (Figure 11) in ethanol (2595 cm^–1^, 2708 cm^–1^), butanol (2615 cm^–1^, 2717 cm^–1^), and hexane (2596 cm^–1^, 2711 cm^–1^).

**Table 4:** Micro-Raman Analysis of the Formation of Amorphous Carbon on LIPSS on Au-Coated Ni, Produced by Femtosecond Laser-Treating Surfaces (*N* = 50, 1 kHz, *E* = 100 μJ) in Ethanol, Butanol, and Hexane

fluid	*I*_D_	*I*_G_	*I*_D_/*I*_G_	fwhm_D_	fwhm_G_
ethanol	414	488	0.82	204	119
butanol	1456	1801	0.80	222	112
hexane	793	909	0.85	229	115

The shape of the 2D band can reveal the number of stacking layers of graphene.^79^ In butanol and hexane, a stacking of 3 graphene layers can be expected. Since the perfect graphite structure was not observed due to the existence of the D-band, it is worth noting that many of these sp^2^ rings were broken due to the laser interaction, thus amorphization and transition from sp^2^ to sp^3^ are highly plausible. Since the GIXRD analysis could not detect crystalline carbon (Figure S2), a low density of crystalline graphite is assumed. Thus, the carbon structures on the laser-treated areas may be analogous to stage 2 of the classification theory of carbon structures by Ferrari and Robertson.^73^ It can be assumed that in the early phase of laser photolysis, graphite nanocrystallites are formed transforming to amorphous carbon when applying consecutive pulses (*N* = 50). Based on Raman spectroscopy, the frequency of the G band (ν_G_) for stage 2 of the carbon structure is within 1600 to 1510 cm^–1^ and *I*_D_/*I*_G_ is between 2 to 0.2,^73^ which agrees with the present measurements (Table 4). Due to carbon amorphization, a broad hump was also observed for ethanol, butanol, and hexane from 2800 to 3000 cm^–1^. This seems related to CH_2_ and CH_3_ vibrations which are both within this spectral region.^80^ A peak at 1042 cm^–1^ in hexane may be related to the vibration of the C–CH_3_ group.^80^

In water, two dispersed D and G peaks at 1401 and 1572 cm^–1^ (Figure 11) are detected. They may indicate the existence of somewhat amorphous surface carbon, since its D-line and G-line intensity are six times lower than for butanol and hexane and 2 times lower than for ethanol. Moreover, the 2D-line in water is quite broad with a very low intensity when compared to alcoholic fluids. The carbon peaks in the pristine areas are very weak with an intensity of about 5 for D-line and G-line (magnified for better visibility). The broad peak from 600 to 1200 cm^–1^ especially in the water area is related to the formation of NiO^81^ on the surface which grows upon laser treatment.

## 4 Conclusions

Self-organized surface structures were produced on Au-coated Ni surfaces by a multipulse femtosecond laser (800 nm) procedure. During the initial period of laser–matter interaction, hydrodynamic instability arises that can be reduced by phase transformations. Thus, the disturbed surfaces release stress and strain via the formation of self-organized structures. The linearly polarized, horizontally aligned femtosecond beam resulted in a vertical orientation of ripples, either as LSFL or HSFL. Fast Fourier transform studies showed deviations in the ripple orientation: HSFL in ethanol, butanol, and hexane exhibited the highest deviation angles from the vertical direction, while in water the deviation was rather small. The maximum HSFL deviation (θ = 24°) was recorded for hexane. The lowest periodicity was evaluated for butanol (280 nm) while the highest one was for hexane (669 nm). Several cubic Au particles were detected for hexane, either between the ripples or on the surface. This may correspond to a femtosecond laser-induced fast cooling rate which assists in freezing the initially small and large ejected molten droplets during the phase explosion and material phase transformation, so that the molten droplets can preserve their primary shapes. Stress confinement on a laser-treated area may also trigger photofragmentation and spallation processes, thus larger particles can be produced.

The random orientation of Ni crystallites and polycrystallinity was detected by spotty diffraction peaks in 2D micro-GIXRD. The highest crystallite size was obtained in butanol, as determined by Williamson–Hall plots. Also, an increase in cell volume and lattice deformation in butanol was due to thermal expansion of the lattice via tensile stresses.

Confocal micro-Raman spectroscopy provided information about graphitization of the laser-treated targets. Photolysis and pyrolysis in carbon-containing solvents cause the formation of byproducts either as graphite networks inside the colloidal solution (e.g., in hexane) or as mostly deposited on the surface as preferentially detected for butanol. However, the existence of a Raman D-band clearly showed that the graphite structures were imperfect. Thus, amorphization occurred due to the applied 50 pulses. This was detected as a broad peak for ethanol, butanol, and hexane from (2800 cm^–1^ to 3000 cm^–1^).

Contact angle measurements of water on the laser-treated areas, together with droplet evaporation as a function of time, showed that all surfaces were rather hydrophilic. Nevertheless, the measured contact angle in all femtosecond laser-treated areas was larger than that of untreated pristine areas. Thus, the effective water-surface interaction is reduced by ripple formation (“lotus effect”). Surfaces femtosecond laser processed in butanol showed the highest contact angle, likely due to more graphite formation when compared to other zones, and due to amorphization of deposited graphite upon consecutive femtosecond pulses. Further development is certainly needed for optimization, but femtosecond laser engineering of surfaces remains a promising route.

## Supporting Information Available

Linear fit of the first 45 seconds of water droplet contact angle measurements of zones laser treated in ethanol, butanol, and water; summary of focal length variations in various liquid media; 1D-μGIXRD and confocal micro-Raman spectroscopy (PDF)
