# Supplementary material for: Formation of Periodic Surface Structures by Multipulse Femtosecond Laser Processing of Au-Coated Ni in Various Fluids
Source: ACS Appl Eng Mater. 2023 Apr 4;1(4):1263–76. doi: 10.1021/acsaenm.3c00070 (PMC10152447; doi:10.1021/acsaenm.3c00070)
Supplement: Supplementary file 1 — em3c00070_si_001.pdf [file em3c00070_si_001.pdf]

Supporting Information

Formation of Periodic Surface Structures by Multipulse Femtosecond Laser Processing of Au-Coated Ni in Various Fluids

Niusha Lasemi,*[1] Gerhard Liedl,[2] and Günther Rupprechter[1]

[1] Institute of Materials Chemistry, Technische Universität Wien, 1060 Wien, Austria

[2] Institute of Production Engineering and Photonic Technologies, Technische Universität Wien, 1060 Wien, Austria

*Email: niusha.lasemi@tuwien.ac.at.

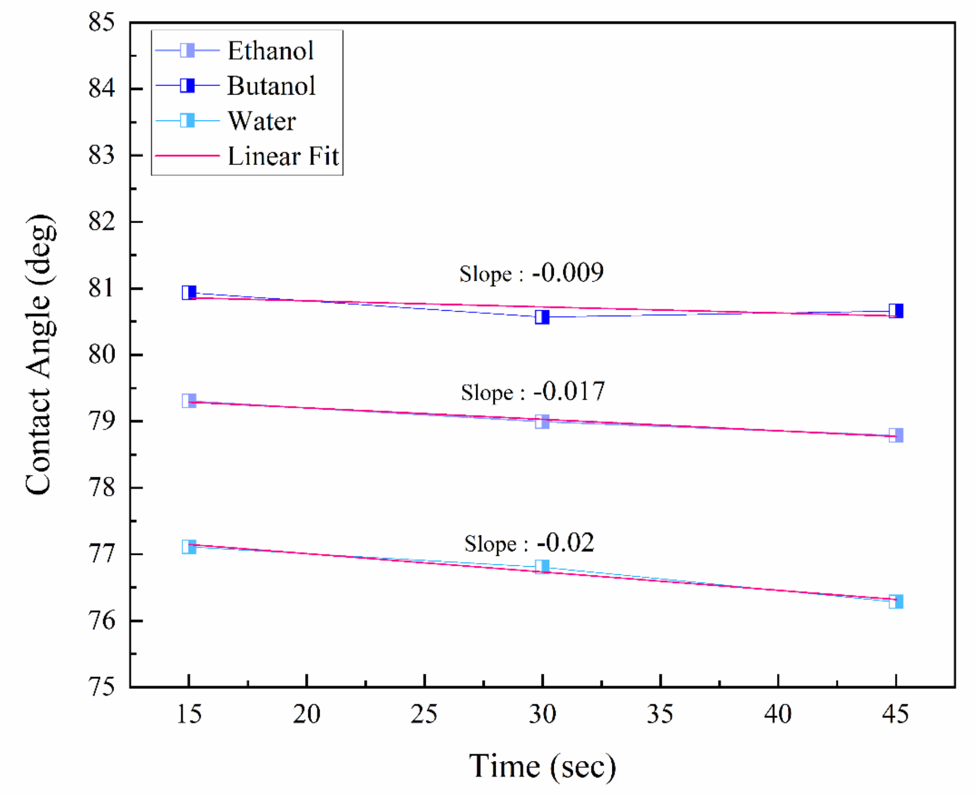

Figure S1. Linear fit of the first 45 seconds of water droplet contact angle measurements of zones laser treated in ethanol, butanol and water.

The equation of $\Delta\tau_{out} = \sqrt{\Delta\tau^2 + \left[4ln2\frac{(\varphi'')}{\Delta\tau}\right]^2}$ was used to calculate the temporal broadening of a Gaussian laser pulse.[55] The initial pulse duration was 30 fs.

Table S1. Calculated final pulse duration ($\Delta\tau_{out}$) in various liquid media. Group velocity dispersion (GVD) and second-order phase coefficient (φ'') were measured for wavelengths (λ) between 700 and 800 nm.

Solvent	GVD (fs^2/mm)	φ'' (fs^2)	$\Delta\tau_{out}$ (fs)
ethanol	42.02	504	55
butanol	65.88	790	78
hexane	62.01	744	75
water	24.90	298	40

The equation of $\Delta f = l(1 - \frac{1}{n})$ was applied to calculate the increased values of focal length (Δf) in various liquids.[64] l is the liquid thickness and n is the refractive index of the solvent.

Table S2. Summary of focal length variations in various liquid media.

Solvent	n	1/n	Δf (mm)
ethanol	1.36	0.735	3.17
butanol	1.39	0.719	3.36
hexane	1.37	0.729	3.24
water	1.33	0.751	2.97

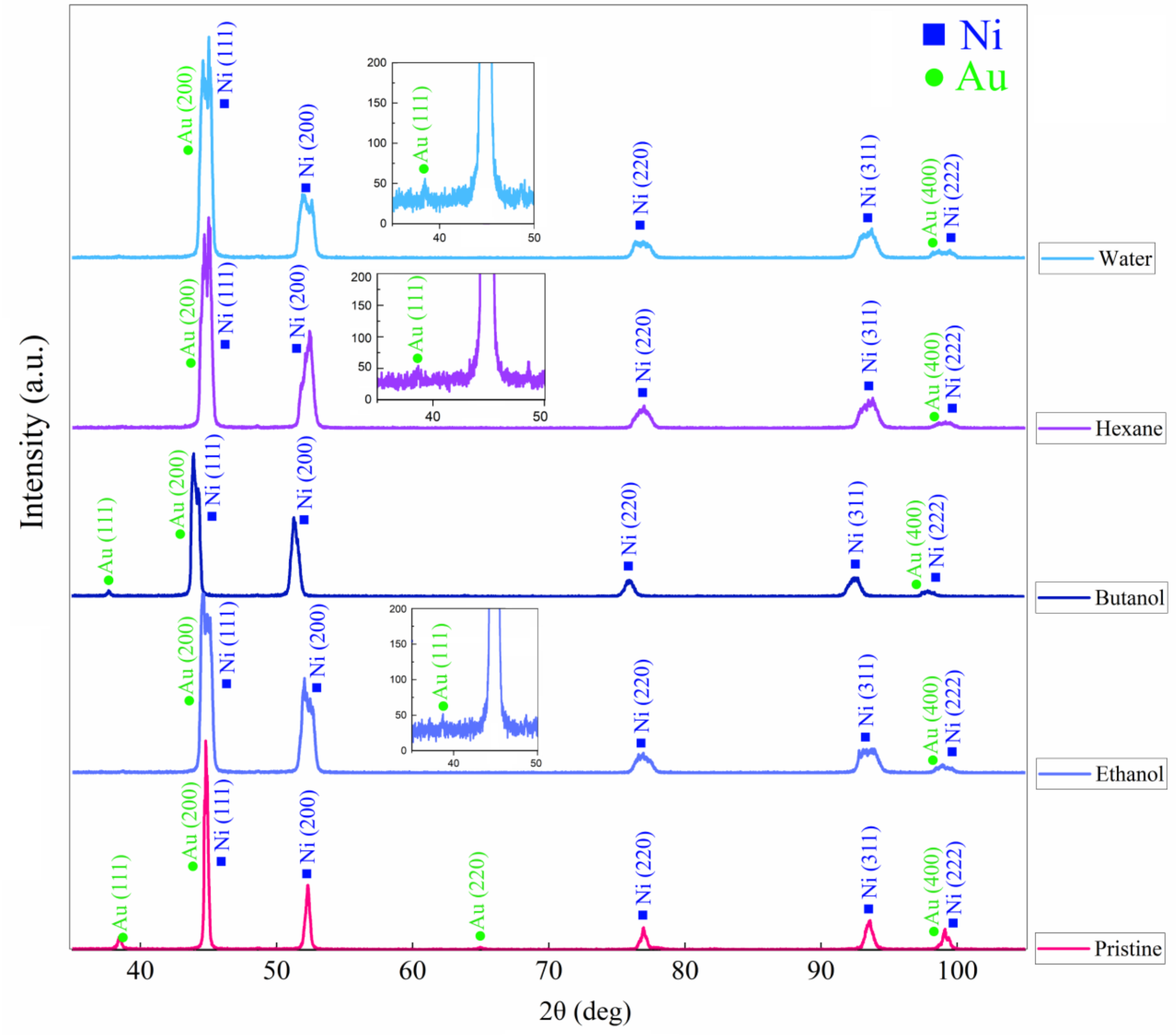

Figure S2. 1D-μGIXRD of pristine and LIPSS zones femtosecond processed in various fluids. Magnified Ni and Au diffraction peaks are shown as insets.

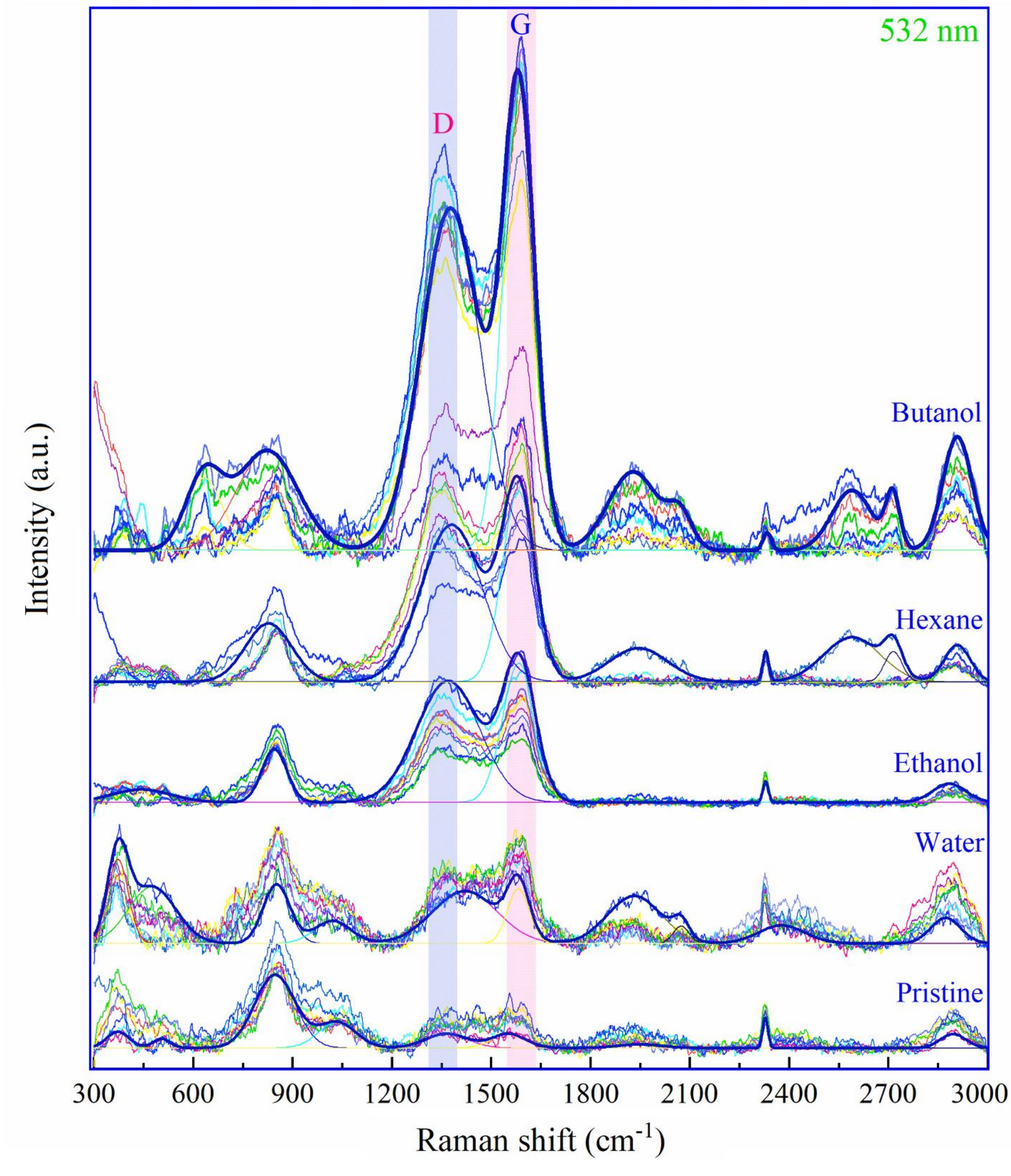

Figure S3. Confocal micro Raman spectroscopy of Au-coated Ni for pristine and LIPSS zones femtosecond processed in various fluids ($N = 50$, 1 kHz, E = 100 μJ). Gauss fit (blue solid line) and respective deconvoluted peaks are selected based on the best R^2. For every fluid, 10 different areas were analyzed.
